# Pioglitazone Attenuates Experimental Colitis-Associated Hyperalgesia through Improving the Intestinal Barrier Dysfunction

**DOI:** 10.1007/s10753-019-01138-3

**Published:** 2020-01-27

**Authors:** Yulin Huang, Chenchen Wang, Xinyu Tian, Yanting Mao, Bailin Hou, Yu’e Sun, Xiaoping Gu, Zhengliang Ma

**Affiliations:** Department of Anaesthesiology, Affiliated Drum Tower Hospital of Medical School of Nanjing University, Nanjing, 210008 Jiangsu province China

**Keywords:** pioglitazone, colitis, chronic pain, hyperalgesia, intestinal barrier

## Abstract

Impaired intestinal mucosal integrity during colitis involves the peroxisome proliferator-activated receptor-γ (PPARγ), an important anti-inflammatory factor in intestinal mucosa homoeostasis, which is a potential target in colitis. Recurrent chronic pain is a vital pathogenetic feature of colitis. Nevertheless, potential functions of PPARγ in the colitis-associated hyperalgesia remain unclear. This study aimed to investigate biological roles of pioglitazone in relieving colitis-associated pain hypersensitivity by a PPARγ tight junction protein-dependent mechanism during the course of dextran sodium sulfate (DSS)-induced intestinal inflammation. The DSS-induced colitis model was generated in C57BL/6 mice. Changes in colitis induced the injury of intestinal mucosal barrier and hyperalgesia after a 6-day treatment of pioglitazone (25 mg/kg, IP injection) were assessed through immunofluorescent, hematoxylin and eosin (H&E) staining, western blot analysis, and determination of paw withdrawal mechanical threshold. A significant reduction of paw withdrawal mechanical threshold occurred after DSS treatment. Follow-up data showed that systematic administration of PPARγ agonist pioglitazone ameliorated the DSS-induced colitis and the development of colitis-associated hyperalgesia by repairing the intestinal mucosal barrier. The tight junction proteins ZO-1 and Claudin-5 were upregulated by PPARγ signaling, which in turn promoted the improvement of intestinal barrier function. Moreover, pioglitazone inhibited phosphorylation of ERK and NF-κB in the colon and decreased the levels of inflammatory cytokines in both colon spine tissues. Furthermore, systemically pioglitazone treatment inhibited the activation of microglia and astrocytes, as well as DSS-induced phosphorylation of NR2B subunit in spinal cord, which was correspondingly consistent with the pain behavior. Pioglitazone ameliorates DSS-induced colitis and attenuates colitis-associated mechanical hyperalgesia, with improving integrity of the intestinal mucosal barrier by directly upregulating tight junction proteins. The PPARγ-tight junction protein signaling might be a potential therapeutic target for the treatment of colitis-associated chronic pain.

## INTRODUCTION

Inflammatory bowel diseases (IBD), including Crohn’s disease and ulcerative colitis, are characterized by diarrhea, weight loss, and chronic pain. IBD result in debilitating illness [1], among which chronic pain [2] emerges from the hyperresponsiveness of neuronal, immune, and endocrine signaling pathways within the intestines, the peripheral [3], and the central nervous system [4]. However, the mechanisms underlying IBD-associated chronic pain are largely unresolved and treatment options are limited. For the nervous system, the mechanisms involved in IBD-associated chronic pain encompass neuronal synaptic changes couple with increased neurotransmitter release [5]. The mechanisms in the inflammatory intestinal wall include interactions of immune cells, macrophages, smooth muscles, and enteric glias [6–9]. Upon epithelial injury and intestinal inflammation in IBD, compromised intestinal barrier integrity subsequently arises, dysregulated transportation of water and ion, exposures of immune cells to bacterial antigens, and triggers reactive enteric gliosis. The above pathological changes eventually result in a profound inflammatory immune response, and in turn, worsening the damage of intestinal mucosa [10, 11]. Tight junctions are composed by a series of transmembrane proteins including the claudins, occludins, junctional adhesion molecules with immunoglobulin-like domains, and intracellular scaffold proteins (*i*.*e*., zonula occludens) [12]. Tight junctions are pivotal in regulating intestinal permeability and maintaining intestinal barrier integrity. Emerging evidences from experimental intestinal inflammation models have supported the idea that a strong connection exists between tight junction protein impairment and intestinal inflammation [11, 13].

Peroxisome proliferator-activated receptor gamma (PPARγ), a number of the nuclear hormone receptor family, exerts a crucial role in mediating inflammatory diseases (*e*.*g*., colitis [14], liver steatosis [15], and rheumatoid arthritis [16]) and inflammatory and neuropathic pain development [17].

The colon expresses a high density of PPARγ, which presents anti-inflammatory effects on inhibiting the activation of NF-κB and expressions of the pro-inflammatory cytokines IL-1 and TNF-α [14, 18]. Earlier studies found PPARγ deficiency alongside elevated activation of NF-κB in the intestinal mucosa in UC patients, suggesting that PPARγ is a potential therapeutic target for UC [14, 19]. As a nuclear receptor, PPARγ-induced sustained changes in gene expressions are widely believed to be the key mechanism of pain reduction [20–22]. Repeated administration of PPARγ agonists reduces neuropathic pain-like behavior and associated molecular changes in the spinal cord dorsal horn [23]. However, it is still unclear how distinct intestinal inflammation contributes to the chronic pain development. Here, we characterized the anti-inflammatory and analgesia effects of systemic PPARγ activation by pioglitazone administration in dextran sodium sulfate (DSS)-induced acute colitis mice [24].

## METHODS

### Animals

Eight-week-old male C57BL/6 mice (Beijing Vital River Laboratory Animal Technology Co., Ltd.) weighing 20 to 25 g at the time of behavioral procedures were housed in a standard environment with a 12-h light/dark cycle, (20 ± 2 °C) temperature and humidity-controlled room with 4 mice per cage. Mice were given free accesses to food and water provided *ad libitum*. All efforts were made to minimize animal suffering, reduce the number of animals used, and use alternatives to *in vivo* techniques. All animal procedures were performed in accordance with the Animal Care Committee of the Institutional Animal Care and Use Committee of the Medical School of Nanjing University.

### Mouse Model and Drug Treatments

For acute colitis, mice (DSS) were given one cycle of 2.5% DSS [25] (MW 36,000–50,000 Da; MP Biomedicals) for 7 days. Twenty mice (CON) and 31 mice (DSS) were used in this study. To target PPARγ in colitis, mice were IP injected with 25 mg/kg body weight [26] of the PPARγ agonist pioglitazone (HY-14601, MCE Chemicals) dissolved in PBS containing 20% DMSO (Sigma Aldrich) during the period of DSS-induced colitis. Control mice were IP injected with an equal volume of PBS containing 20% DMSO. Seven mice (CON + Veh), 21 mice (DSS + Veh), and 17 mice (DSS + Pio) were used in the study. During the course of the experiment, mice were monitored for body weight, diarrhea, and macroscopic bleeding.

#### Paw Withdrawal Mechanical Threshold

Paw withdrawal mechanical threshold (PWMT) determined at day 1 was recorded as the baseline. In addition, PWMT was performed every day during DSS treatment or pioglitazone injection, and every other day till day 21. All tests were performed during the light phase. Before determination, mice were allowed to acclimatize for at least 30 min. PWMT was performed in a quiet test room by the same investigator. Von Frey filaments (Stoelting, Wood Dale, IL, USA) were used to assess the mechanical allodynia as previously reported [27]. Briefly, the mice were placed into individual transparent compartments onto a metal mesh floor. Different von Frey filaments (0.16, 0.4, 0.6, 1.0, 1.4, and 2.0 g) were applied to the hind paw. The filaments were pressed vertically against the plantar surface with sufficient force to cause a slight bending against the paw for 6 to 8 s. An interval for at least 10 min was necessary between the two stimulations. Brisk withdrawal of the paw or paw flinching was regarded as a positive response. Each mouse was tested five times per stimulus strength. The lowest von Frey filament stimulus strength that produced at least 3 positive responses was recorded as the reasonable paw withdrawal mechanical threshold.

### Western Blot

Total tissue or cell lysates were prepared with a detergent lysis buffer. Western blot was performed using the indicated primary antibodies: NF-κB p65 (1:1000, Cell Signaling Technology, no. 8242, MA, USA), phospho-p65 (Ser536) (1:1000, Cell Signaling Technology, no. 3031, MA, USA), ERK (1:1000, Cell Signaling Technology, no. 9102, MA, USA), phospho-ERK (p44/p42) (Thr202/Tyr204) (1:1000, Cell Signaling Technology, no. 9102, MA, USA), GFAP (1:1000, Cell Signaling Technology, no. 3670, MA, USA), MMP9 (1:1000, Abcam, no. 38898, Cambridge, UK), NR2B (1:1000, Abcam, no. 65783, Cambridge, UK), phospho-NR2B (1:1000, Abcam, no. 3856, Cambridge, UK), IL-6 (1:1000, Abcam, no. 208113, Cambridge, UK), TNF-α (1:500, Santa Cruz Biotechnology, sc52746, Santa Cruz, CA), Iba1 (1:500, Santa Cruz Biotechnology, sc32725, Santa Cruz, CA), ZO-1 (1:1000, Thermo Fisher, no. 617300, US), Claudin-5 (1:1000, Thermo Fisher, no. 35-2500, US), PPARγ (1:1000, Proteintech, 16643-1-AP, US), anti-GAPDH (1:1000, Boster Biotechnology, BM1985, Wuhan, China), and α-tubulin (1:1000, Boster Biotechnology, BM3885, Wuhan, China). Each blot was repeated three times.

### Immunofluorescence

Immunofluorescence was performed as previously reported. Briefly, the lumbar segments of the spinal cords were extracted and post fixed in 4% paraformaldehyde, followed by dehydration in 30% sucrose at 4 °C. Serial frozen sections were cut in a freezing microtome into 20-μm thick slides. The following indicated primary antibodies were used: Iba-1 (1:500, Wako, 016-26721, Japan) and GFAP (1:100, Cell Signaling Technology, no. 3670, MA, USA). The secondary antibodies used in this study included the following: goat anti-rabbit (1:3000, Alexa 488-conjugated, ThermoFisher, A32723, Waltham, MA) and goat anti-mouse (1:3000, Alexa 594-conjugated, A32740, ThermoFisher, Waltham, MA). DAPI (Abcam, Cambridge, no. 104139, UK) staining was used to determine the cell nuclei.

### Histopathological Analysis

Ten percent buffered neutral formalin-embedded colon sections (5 μm) were stained with hematoxylin and eosin (H&E) and independently analyzed by pathologists in a blinded way.

### Statistical Analysis

Data were expressed as the mean ± SE. SPSS 22.0 (SPSS Inc., Chicago, IL) was used to conduct all the statistical analyses. Mice were assigned to different treatment groups in a randomized manner. Multiple comparisons were carried out to determine the overall differences of pain behaviors at each time point. Repeated measures analysis of variance (ANOVA) was performed to assess the changes of pain behaviors over time. One-way ANOVA was used to determine differences in the results of colon length and immunofluorescence among groups.

In both cases, when significant main effects were observed, Bonferroni *post hoc* tests were conducted to determine the source(s) of these differences. *P* < 0.05 was considered statistically significant.

## RESULTS

### Downregulated PPARγ, Activated P65, and ERK Accompanied Release of Inflammatory Cytokines in Colonic Tissues of DSS-Induced Colitis Mice

**Fig. 1:**
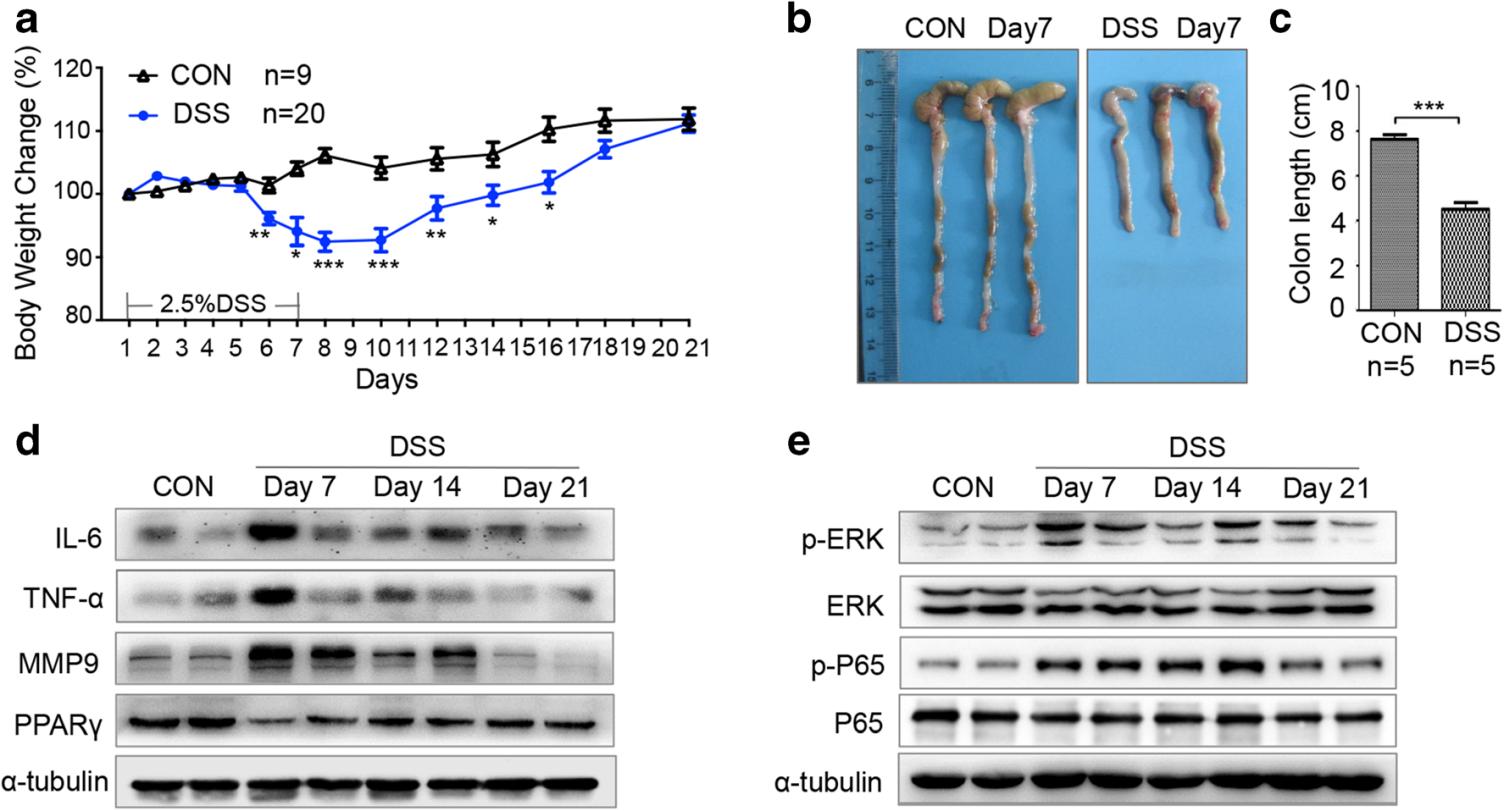
PPARγ expression is impaired during colitis with activation of ERK and P65. **a** Percentage of weight change during 2.5% DSS treatment and recovery. **b** Image of colon in 2.5% DSS-treated mice and control mice on day 7. **c** Quantification of colon length. **d** Increased IL-6, TNF-α, and MMP9 expression in inflamed colonic tissue of 2.5% DSS-treated mice on day 7 and day 14. Decreasing PPARγ expression on day 7. **e** Increased ERK and P65 activation in inflamed colonic tissue after DSS treatment. Immunoblot analysis is performed in tissue lysates with indicated antibodies. Each lane represents one mouse. *p < .05, **p < 0.01, ***p < 0.001 compared with CON and DSS group mice. Error bars represent ± SE.

To explore regulatory effect of PPARγ on the colon with intestinal inflammation, C57BL/6 mice were treated by 2.5% DSS for 7 days. DSS-treated mice showed more pronounced weight loss than that of control mice (Fig. 1a). Acutely DSS-treated mice also showed a significant change in colon length (Fig. 1b, c). Colonic levels of the inflammatory cytokines TNF-α and IL-6 as markers of disease activity were measured by using western blot, which were significantly upregulated in the acute phase of colitis (day 7). Then, the expressions of TNF-α and IL-6 continued to increase in early recovery phase (day 14) and decreased later on day 21. A previous study showed that MMP9 was highly expressed in UC patients compared with control tissues [28]. Thus, we detected the expression of MMP9 in colonic tissues. MMP9 was notably upregulated after DSS treatment on day 7 and day 14. Western blot analysis also showed that DSS treatment notably reduced PPARγ expression in day 7 (Fig. 1d). Consistent with the levels of TNF-α and IL-6, further analysis showed that P65 and ERK were strongly activated in DSS-treated mice on day 7 and day 14 (Fig. 1e). Studies indicated that PPARγ has been identified to activate the NF-κB pathway and MAPK pathway, thus decreasing the levels of pro-inflammatory cytokines [29, 30]. These results suggested that PPARγ in the colon participates in the progression of acute intestinal inflammation.

### Tight Junction Protein Impairment Alone with Activation of P65 and ERK

**Fig. 2:**
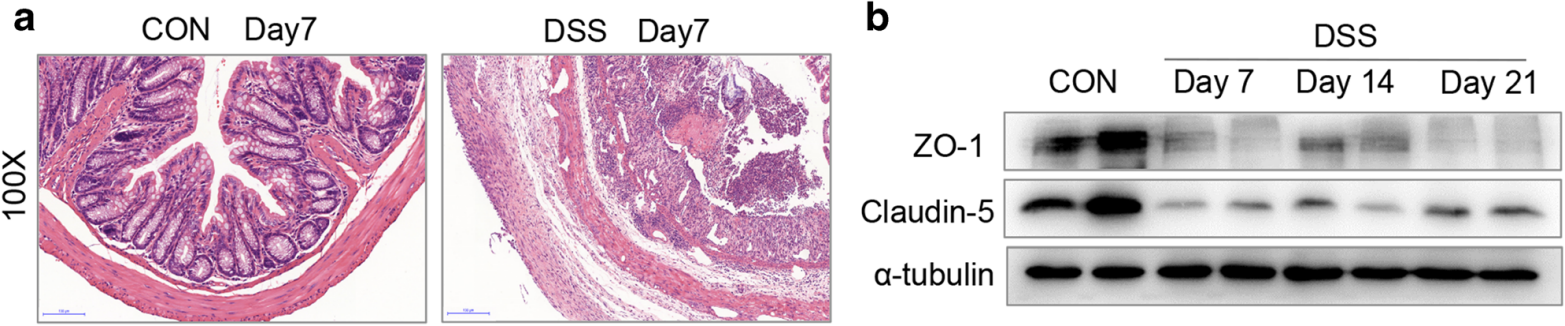
Gut epithelial barrier dysfunction accompanied with decreased the expression of ZO-1 and claudin-5 in colitis. **a** H&E staining of a section obtained from the distal colons of CON and DSS-treated mice on day 7. **b** Decreased expression of ZO-1 and claudin-5 after DSS treatment. Each lane represents one mouse.

Histological changes on day 7 of acute DSS colitis were analyzed by H&E staining of paraffin-embedded colonic cross-sections. The results showed strong transmural inflammation with loss of crypt structure, severe epithelial erosions, and more neutrophilic infiltrates in DSS-treated mice. H&E analysis indicated a breakdown of epithelial barrier function after DSS treatment (Fig. 2a). It is reported that impairment of epithelial tight junction proteins is responsible for decreased barrier integrity leading to colitis [31]. ZO-1 and claudin-5 are important epithelial tight junction proteins [32]. Western blot analysis showed that the expressions of ZO-1 and claudin-5 were significantly downregulated in DSS-induced colitis mice compared with the control group on day 7, day 14, and day 21 (Fig. 2b), suggesting the dysfunction of epithelial barrier.

### Acute Colonic Inflammation Leads to Persistent Pain, Increased Inflammatory Cytokines, and Phosphorylation of NR2B in the Spinal Cord

**Fig. 3:**
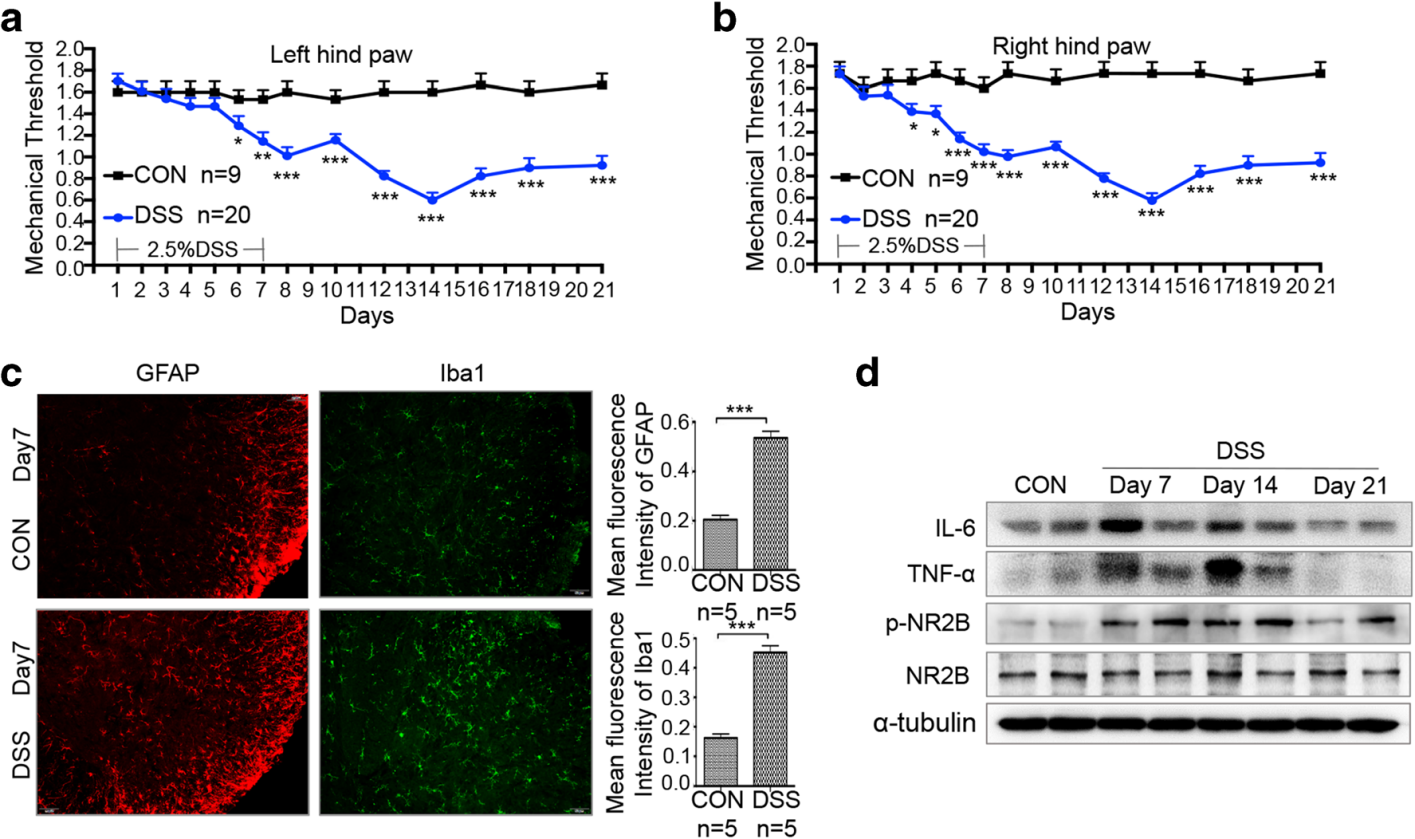
Activation of astrocyte and microglia as well as NR2B signaling is required for colitis-reduced mechanical threshold. **a** Paw withdrawal mechanical threshold in response to von Frey filaments in CON and DSS-treated mice from day 1 to day 21. **b** Activation of spinal microglia and astrocyte. Immunofluorescent staining and corresponding quantification of staining intensity of Iba-1 (a microglia marker) and GFAP (an astrocyte marker) in the spinal cords performing in CON and DSS (day 7) group mice. **c** Increased expression of IL-6, TNF-α, and p-NR2B after DSS treatment. Spinal cord lysates of CON and DSS (day 7, day 14, and day 21) group mice. Each lane represents one mouse. All data are presented as the mean ± SE. *p < 0.05, **p < 0.01, and ***p < 0.001 compared with CON and DSS group mice.

Chronic abdominal pain frequently happens in patients with inflammatory bowel disease [33]. Persistent abdominal pain also occurs in mice with experimental acute intestinal inflammation [34]. However, whether acute intestinal inflammation would result in hyperalgesia remains unclear. Thus, PWMT was performed to evaluate mechanical allodynia and hyperalgesia. After DSS treatment, the withdrawal threshold of both hind paws to mechanical stimuli was lower in experimental mice compared with control groups. Withdrawal thresholds in DSS-induced colitis mice significantly decreased between day 6 and day 21 (Fig. 3a, b), indicating the persistent discomfort even though body weight gradually recovered from day 10. These data indicated that DSS-induced acute colitis would lead to inflammation-related hyperalgesia.

DSS treatment resulted in evident activation of microglia and astrocytes with increased levels of inflammatory cytokines TNF-α and IL-6 in the lumbar spinal cord (Fig. 3b, c). In addition, phosphorylation and activation of NR2B in the spine are vital in the maintenance of central sensitization and mechanical allodynia [35, 36]. Results of western blot showed that DSS treatment notably increased phosphorylation of NR2B in the spinal cord from day 7 to day 21, which was consistent with the changes of PWMT (Fig. 3c).

### Pioglitazone Alleviates DSS-Induced Immune Response Through Downregulation of P65 and ERK

**Fig. 4:**
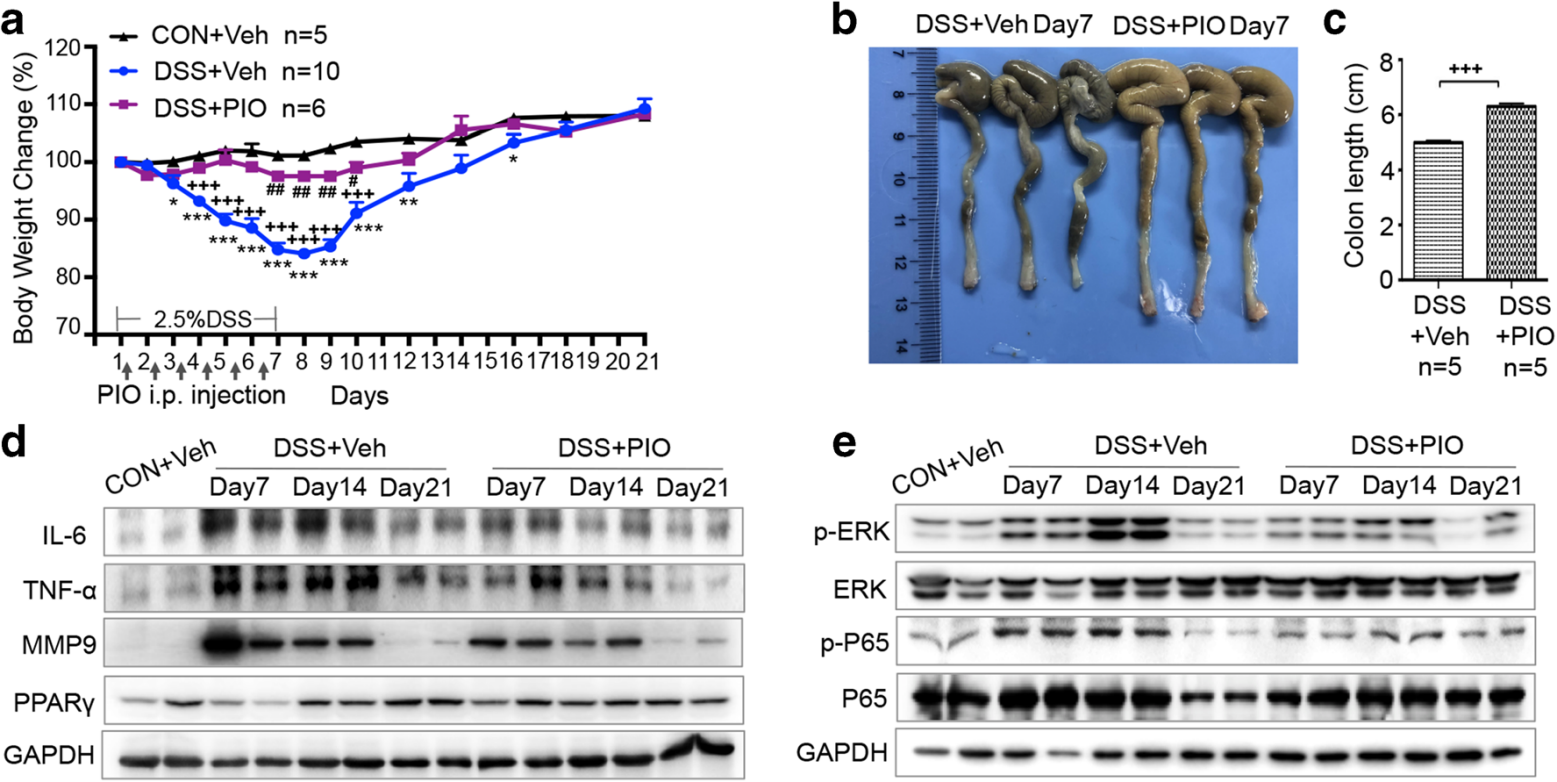
Pioglitazone promoted the recovery from intestinal inflammation. **a** Percentage of weight change in indicated groups. Daily IP injection of PIO during DSS administration. **b** Image of colons collected from DSS + Veh and DSS + PIO group mice (day 7). **c** Quantification of colon lengths in the different groups. **d** Colonic lysates of CON + Veh, DSS + Veh, and DSS + PIO (day 7, day 14, and day 21) group mice were analyzed by immunoblotting with IL-6, TNF-α, MMP9, and PPARγ antibodies, respectively. Each lane represents one mouse. **d** Colonic lysates of CON + Veh, DSS + Veh, and DSS + PIO (day 7, day 14, and day 21) group mice were analyzed by immunoblotting with p-ERK, ERK, p-P65, and P65 antibodies, respectively. Each lane represents one mouse. All data are presented as the mean ± SE. *p < 0.05, **p < 0.01, and ***p < 0.001 compared with CON + Veh and DSS + PIO group mice. ^#^*p* < 0.05, ^##^*p* < 0.01, ^###^*p* < 0.001 compared with CON + Veh and DSS + Veh group mice. ^+^*p* < 0.05, ^++^*p* < 0.01, ^+++^*p* < 0.001 compared with DSS + Veh and DSS + PIO group mice.

PPARγ, a member of the nuclear hormone receptor family, can be activated by anti-diabetic thiazolidinedione drugs, such as pioglitazone [37]. The treatment with pioglitazone or vehicle was introduced for 7 days during DSS administration. Mice with DSS-induced colitis receiving vehicle presented more pronounced weight loss, shorter length of colons, and more severe histologic alterations and macroscopic damages compared with those of controls. In the preventive group, pioglitazone successfully protected against weight loss and colonic shortening (Fig. 4b, c). Obviously, pioglitazone rescued DSS-induced reduction of PPARγ in colon tissues (Fig. 4d). A previous research has shown that PPARγ could regulate the NF-κB pathway and MAPK signaling activation, thus decreasing the levels of pro-inflammatory cytokines [38]. Western blot analysis showed that DSS-induced activation of P65 and ERK was significantly inhibited in pioglitazone preventive group, along with decreased expressions of IL-6, TNF-α, and MMP9 (Fig. 4d, e).

### Enhanced Tight Junction Expression and Recovered Intestinal Barrier Function Induced by Pioglitazone

**Fig. 5:**
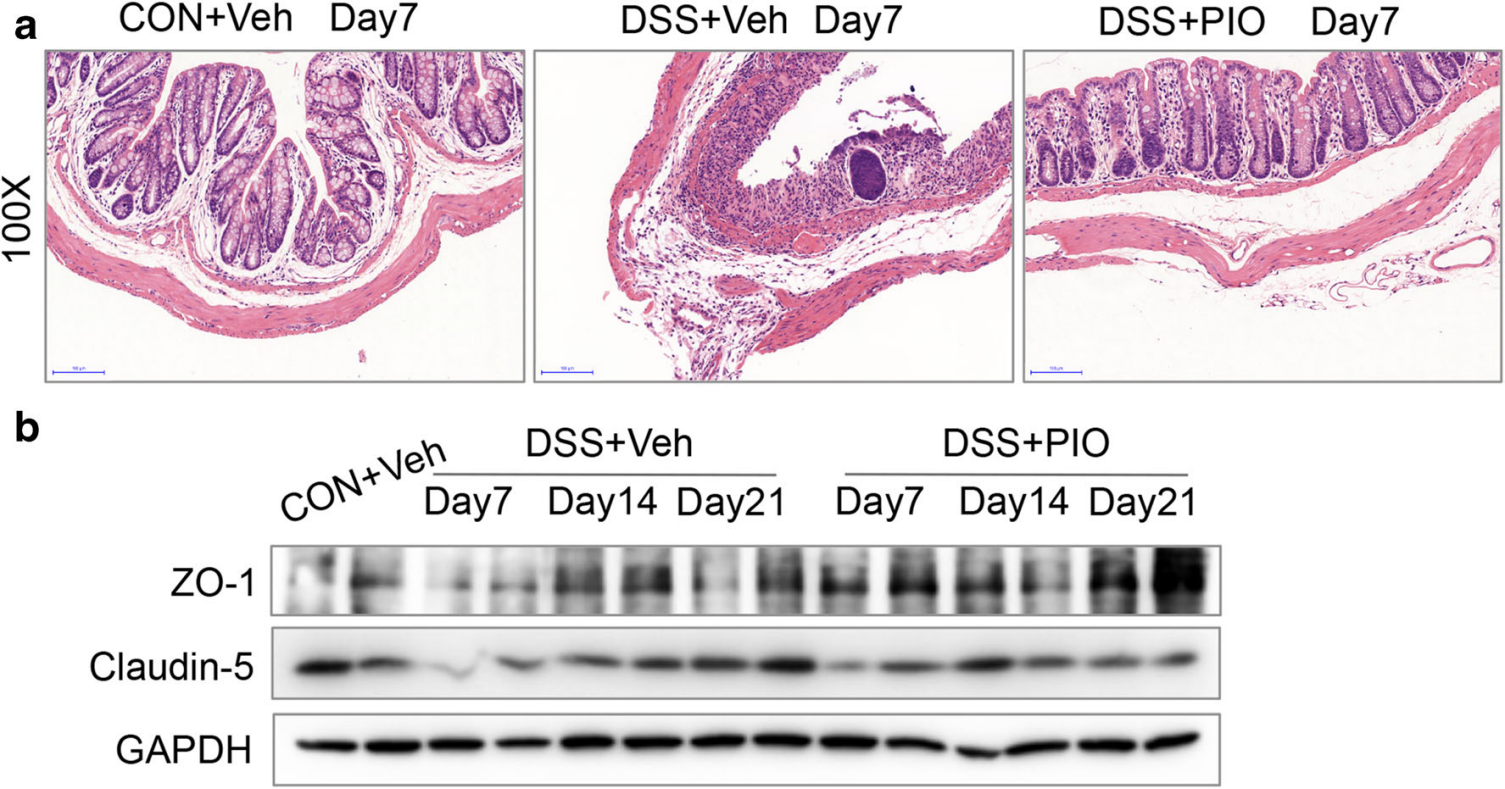
Pioglitazone-alleviated tissue injury was histologically evident by increasing the expression of ZO-1 and claudin-5. **a** H&E staining of a section obtained from the distal colons of CON + Veh, DSS + Veh, and DSS + PIO mice on day 7. **b** Colonic lysates of CON + Veh, DSS + Veh, and DSS + PIO (day 7, day 14, and day 21) group mice were analyzed by immunoblotting with ZO-1 and claudin5 antibodies, respectively. Each lane represents one mouse.

Pathological injury in mouse colorectum was independently examined by experienced pathologists. H&E staining (magnification 100×) of colorectum tissues from 3 mice in each group was conducted on day 7 (Fig. 5a). Within intestinal samples from mice in each group, expressions of claudin-5 and ZO-1 were significantly downregulated in the intestines of DSS-treated mice, whereas the expression levels of them were dramatically elevated in the intestines of the pioglitazone preventive group (Fig. 5b).

### Pioglitazone Reduces Mechanical Hypersensitivity in Colitis Mice

**Fig. 6:**
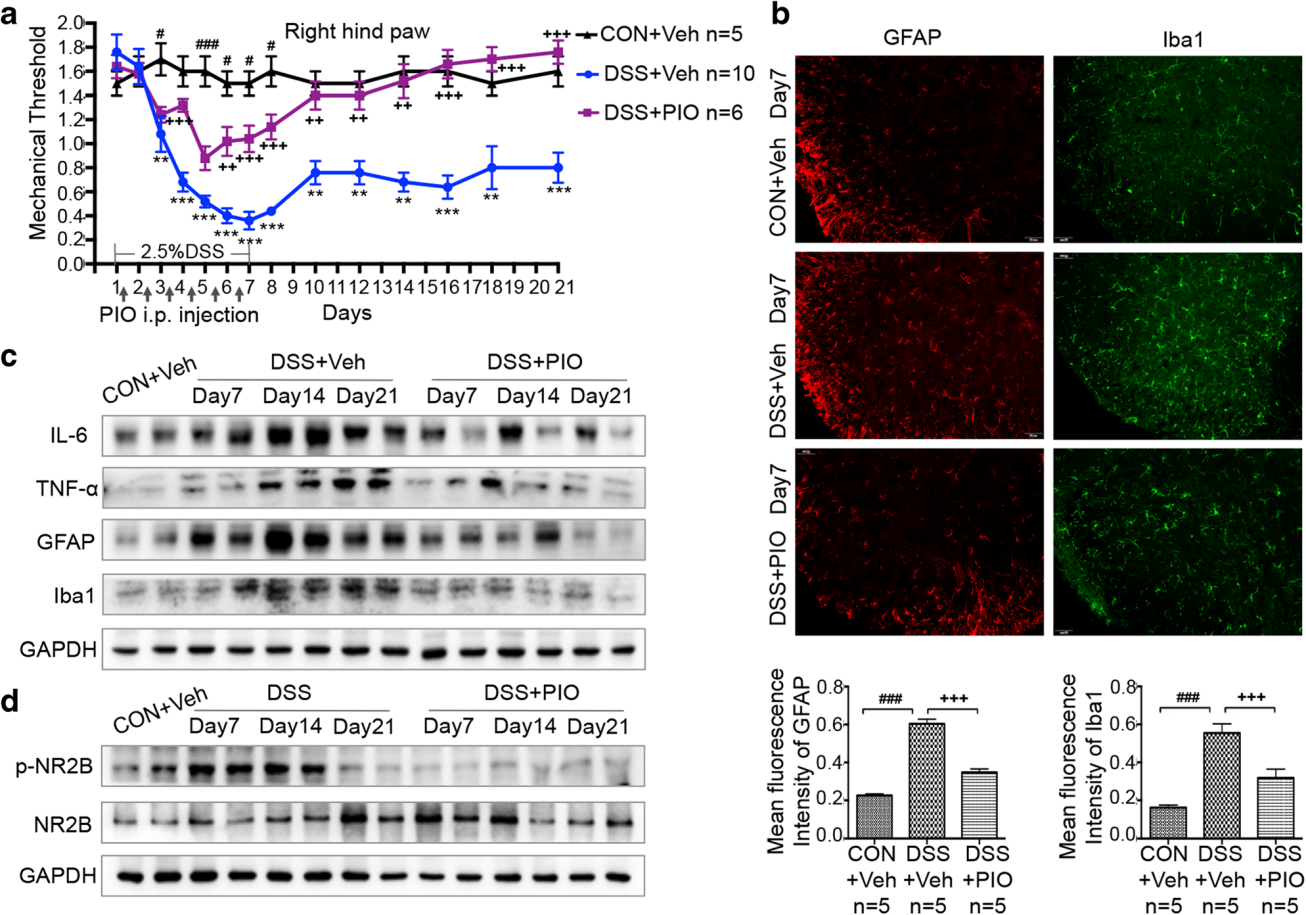
The antihyperalgesic effect of pioglitazone is mediated by inhibiting colitis-induced spinal inflammation. **a** Paw withdrawal mechanical threshold in response to von Frey filaments in indicated groups (CON + Veh, DSS + Veh, and DSS + PIO) from day 1 to day 21. **b** Immunofluorescent staining and corresponding quantification of staining intensity of Iba-1 (a microglia marker) and GFAP (an astrocyte marker) in the spinal cords in indicated groups (CON + Veh, DSS + Veh, and DSS + PIO) on day 7. **c** Spinal cord lysates of CON + Veh, DSS + Veh, and DSS + PIO (day 7, day 14, and day 21) group mice were analyzed by immunoblotting with IL-6, TNF-α, GFAP, and Iba1 antibodies. **d** Immunoblotting was performed to analyze the expressions of p-NR2B and NR2B in spinal cord of CON + Veh, DSS + Veh, and DSS + PIO (day 7, day 14, and day 21) group mice. All data are presented as the mean ± SE. *p < 0.05, **p < 0.01, and ***p < 0.001 compared with CON + Veh and DSS + PIO group mice. ^#^*p* < 0.05, ^##^*p* < 0.01, ^###^*p* < 0.001 compared with CON + Veh and DSS + Veh group mice. ^+^*p* < 0.05, ^++^*p* < 0.01, ^+++^*p* < 0.001 compared with DSS + Veh and DSS + PIO group mice.

Pioglitazone-treated mice showed decreased mechanically hypersensitivity and gradual recovery of inflammation during 2 weeks post-DSS discontinuation (Fig. 6a). Less activated microglia and astrocytes in spinal dorsal cord could also be observed in pioglitazone-treated mice compared with DSS-treated mice, which were consistent with protein level changes of Iba1 and GFAP in the spinal dorsal cord (Fig. 6b, c). Western blot analysis of spinal dorsal cord also showed that pioglitazone treatment remarkably decreased the levels of TNF-α and IL-6 (Fig. 6c). Further analysis showed that phosphorylation of NR2B was notably suppressed after pioglitazone intervention, which was in accordance with the findings of pain-related behaviors (Fig. 6d).

## DISCUSSION

This study demonstrated that systematic administration of pioglitazone, an agonist of PPARγ, could alleviate DSS-induced colitis, attenuate colitis-associated mechanical hyperalgesia, and improve integrity of the intestinal mucosal barrier by directly upregulating tight junction proteins.

Increasing effects have been made on clarifying the possible role of gut-brain-axis in the complex regulation of pain [39, 40]. However, it is still necessary to explore more crosstalks between the intestinal tract and the central nervous system or enteric nervous system in pain regulation. The etiology of IBD-associated chronic pain remains enigmatic. Visceral hypersensitivity and systematic mechanical hyperalgesia have been well explored in the researches of IBD-associated pain [41]. Hypersensitivity is driven by peripheral and central mechanisms [42], involving the participation of the intestinal wall, spinal cord, and brain centers [43]. Nonetheless, contemporary histopathology studies in IBD patients, as well as the established murine models of intestinal inflammation, implicate a causative combination of progressive destruction of the intestinal mucosal barrier and altered mucosal immune responses [44].

Previous studies have indicated a potential linkage between genetic mutations or deletions in tight junction-associated proteins and development of IBD [45]. Further studies also have proven that stabilizing junctional complex in intestinal barrier could attenuate intestinal inflammation [46]. In this paper, tight junction proteins claudin-5 and ZO-1 were notably downregulated in mice after DSS treatment, and further H&E identically supported our findings, indicating the impairment of intestinal mucosal barrier in DSS-induced colitis mice. Interestingly, decreased protein level of PPARγ was corresponding to the severity of intestinal inflammation. Based on the differences in mechanical thresholds observed between controls and experimental mice, potential correlation between intestinal inflammation and changes in mechanical hypersensitivity was investigated. Because the PPARγ system is a relevant target for the treatment of inflammatory diseases and pain, molecular mechanisms underlying the healing of intestinal mucosal injury and analgesic effect of pioglitazone in DSS-induced colitis mice were clarified here.

In our analysis, the PPARγ antagonist pioglitazone was administered once a day in mice from day 1 to day 7 alone or in combination with DSS. Our data suggested that the anti-inflammatory effects of pioglitazone were closely linked to its regulatory ability on tight junction protein expressions. The prevention of intestinal mucosal barrier injury observed in pioglitazone-treated mice with colitis was also associated with increased expressions of tight junction proteins and reduced protein levels of TNF-α, IL-6, and MMP9, which are soluble mediators controlled by the ERK-NF-κB [38]. However, the reduction of these inflammatory mediators in colon tissue could be simply a consequence of a decreased infiltration of neutrophils, which were beneficial from recovered intestinal mucosal barrier after pioglitazone treatment. Our data also demonstrated a marked inhibition of the p65 NF-κB subunit and ERK in the colons of pioglitazone-treated mice with colitis. It is possible that impaired intestinal mucosal barrier allowed the passage of inflammatory mediators in DSS-treated mice, contributing to the systemically immune-mediated activation. In the current study, relative levels of TNF-α and IL-6 in mouse spinal cord after DSS treatment were significantly enhanced. It is well known that multiple inflammatory mediators could activate microglia and astrocytes [47], which maintain central sensitization and mechanical hyperalgesia [48]. In addition, activation of NMDA receptor in the spinal cord dorsal horn is one of key events, driving central sensitization and pain hypersensitivity [49]. Our results showed a significant colitis-induced activation of NMDA receptor-NR2B subunit, which is of importance role in regulating spinal synaptic plasticity in persistent pain conditions. In our study, systemically pioglitazone treatment inhibited the activation of microglia and astrocytes, and DSS-induced phosphorylation of NR2B subunit in the spinal cord, thus contributing to pain relief.

## LIMITATIONS

In the present study, there are several limitations. Firstly, our results are not the first to describe the anti-inflammatory effect of pioglitazone on colitis. Secondly, although it has been reported that intrathecal or systematical of pioglitazone attenuates hyperalgesia in a neuropathic pain model [20, 50]. However, we only observe the effects of systematical administration of pioglitazone on colitis and related pain hypersensitivity. Thirdly, in fact, there are much more possible mechanisms of colitis-associated chronic pain. Further evidences indicated that colitis-associated pain can result from sympathetic nerves to the spinal cord *via* the dorsal roots [51]. Except for intestinal mucosa injury, the commensal microbiota-gut-brain axis has been found to be both ecologically and functionally perturbed in colitis and related pain [52]. The pain processing of colitis patients with abdominal pain is tightly associated with gut-derived neurochemical metabolites [53]. A further study on the specific mechanism of brain-gut-network in colitis-associated pain has been conducted in our laboratory.

## CONCLUSIONS

In summary, we demonstrated that systemic administration of pioglitazone markedly alleviated pain hypersensitivity by decreasing inflammatory mediators coming into the spinal cord from the injured intestinal mucosa in DSS-induced acute colitis. Pioglitazone can ameliorate colitis-associated intestinal barrier function by upregulating tight junction proteins, which may result in the reduction of spinal levels of inflammatory mediators. We proposed that the PPARγ tight junction protein signaling might be a potential therapeutic target for the treatment of colitis-associated chronic pain.

## Abbreviations

PPARγ: proliferator-activated receptor-γ
DSS: dextran sodium sulfate
PWMT: paw withdrawal mechanical threshold
IBD: inflammatory bowel disease
H&E: hematoxylin and eosin
